# Effect of Different Surface Treatments on Titanium Dental Implant Micro-Morphology

**DOI:** 10.3390/ma12050733

**Published:** 2019-03-04

**Authors:** Gaetano Marenzi, Filomena Impero, Fabio Scherillo, Josè Camilla Sammartino, Antonino Squillace, Gianrico Spagnuolo

**Affiliations:** 1 Department of Neurosciences, Reproductive and Odontostomatological Sciences, University of Naples “Federico II”, Via Pansini 5, 80131 Naples, Italy; gianrico.spagnuolo@gmail.com; 2 Department of Chemical, Materials and Industrial Production Engineering, University of Naples “Federico II”, P.le Tecchio 80, 80125 Napoli, Italy; filomena.impero@unina.it (F.I.); fabio.scherillo@unina.it (F.S.); squillac@unina.it (A.S.); 3 Department of Biology and Biotechnology “L. Spallanzani”, University of Pavia, Via Ferrata 1, 27100 Pavia, Italy; jose.sammartino@iusspavia.it; 4 Institute of Dentistry, I. M. Sechenov First Moscow State Medical University, 119146 Moscow, Russia

**Keywords:** dental implant, titanium, Ti6Al4V, surface morphology, treatments

## Abstract

Background: Titanium dental implants are today widely used with osseointegration mainly dependently on the implant surface properties. Different processing routes lead to different surface characteristics resulting, of course, in different in situ behaviors of the implants. Materials: The effect of different treatments, whether mechanical or chemical, on the surface morphology of titanium implants were investigated. To this aim, various experimental methods, including roughness analysis as well scanning electron microscope (SEM) observations, were applied. Results: The results showed that, in contrast to the mechanical treatments, the chemical ones gave rise to a more irregular surface. SEM observations suggested that where commercial pure titanium was used, the chemical treatments provided implant surfaces without contaminations. In contrast, sandblasted implants could cause potential risks of surface contamination because of the presence of blasting particles remnants. Conclusions: The examined implant surfaces showed different roughness levels in relation to the superficial treatment applied. The acid-etched surfaces were characterized by the presence of deeper valleys and higher peaks than the sandblasted surfaces. For this reason, acid-etched surfaces can be more easily damaged by the stress produced by the peri-implant bone during surgical implant placement.

## 1. Introduction

Many studies have reported that the bone–implant interface can influence peri-implant bone healing and the osseointegration process [1,2,3,4,5,6,7,8,9,10]. Not only does a rough implant surface ensure an improved bone anchorage when compared to a smooth implant surface, but it also promotes mesenchymal cell differentiation toward an osteoblastic phenotype [11]. A large number of investigations have even revealed that the most favorable bone responses are obtained with a moderately micro-roughened profile with an arithmetic mean deviation of the roughness profile (R_a_) ranging between 1.0 and 2.0 μm. However, an optimal roughness and superficial morphology are still controversial and need to be more clearly defined [1,2,6,12]. In order to increase the contact area between the living bone and the implant and thus enhance osseointegration, manufacturers have introduced many treatments, both chemical (acid-etching) and mechanical (grit-blasting) or a combination of the two [13]. Rough titanium implant surfaces can be obtained by means of addition (titanium plasma spray), subtraction (acid-etching. grit-blasting), or mixed techniques [9,13]. Acid-etching is the most common chemical method used for dental surface treatment. Acid-etching is often performed using hydrofluoric (HF), nitric (HNO_3_), sulfuric (H_2_SO_4_) acids, and combinations thereof [14]. The resultant surface characteristics may vary in relation to time, type, and concentration of the etching acid. The chemically etched implants produced morphologies characterized by a large fractal dimension and punching surfaces that helped cell proliferation [15].

Grit-blasting is a common physical method used for surface treatments; it is performed by a projection of silica, hydroxyapatite, alumina, and TiO_2_ particles ranging in size between 25 and 75 μm [16]. Many parameters such as temperature, pressure, type, and size of the blasting particles may have an impact on the final result [14]. The alumina remains and the blasting-modified surface energy of the implants encourage cell adhesion, but impede cell growth.

In order to improve the roughness of the surface, many industrial techniques have also been developed to produce a thin coat (sol–gel deposition, sputtering coating techniques, or ion beam-assisted deposition) [17,18]. 

Each industrial method is able to produce different implant surfaces by modifying the treatment parameters; however, the fixture chemical composition can also influence the surface characteristics. 

Generally, dental implants are made of two different types of titanium: Grade 4 titanium, which is also known as commercially pure titanium (CPTi), and a Grade 5 titanium alloy [19]. Because of their different and mainly mechanical characteristics such as hardness, dental implants from these materials have to be treated according to different procedures in order to obtain surface roughness. Dental implants obtained from CPTi have to be chemically etched, whilst the ones made of a Grade 5 titanium alloy can be sandblasted. These two different treatments result in implant surfaces with very different characteristics [9,19]. 

Even though surface roughness seems to favor peri-implant bone healing in different ways, many authors have revealed that the insertion of a rough fixture leads to a greater titanium wear than in smooth surfaces [20,21,22,23,24,25]. The real effects of metal and surface contaminant release during the osseointegration process are not yet fully understood, even if they are reported as a possible disturbing factor in the process of bone remodeling balance [26,27,28,29].

The aim of this study was to analyze the chemical composition and microstructural conformation of dental implants subjected to different surface treatments, thus evaluating the predisposition to titanium wear during their surgical placement. 

## 2. Materials and Methods 

Two dental implants made by Biotech Dental (Kontact and Kontakt S, Biotech Dental, Salon-de-Provence, France) with different macro-morphology and surface treatments were selected for this research. For each fixture, two different dimensions (3.6 mm and 4.2 mm) were analyzed. The main characteristics of the selected implants are reported in Table 1. 

The chemical etching was performed using a solution made of 2% HF (Biotech Dental, Salon-de-Provence, France) and 10% HNO_3_ (Biotech Dental, Salon-de-Provence, France).

**Figure 1:**
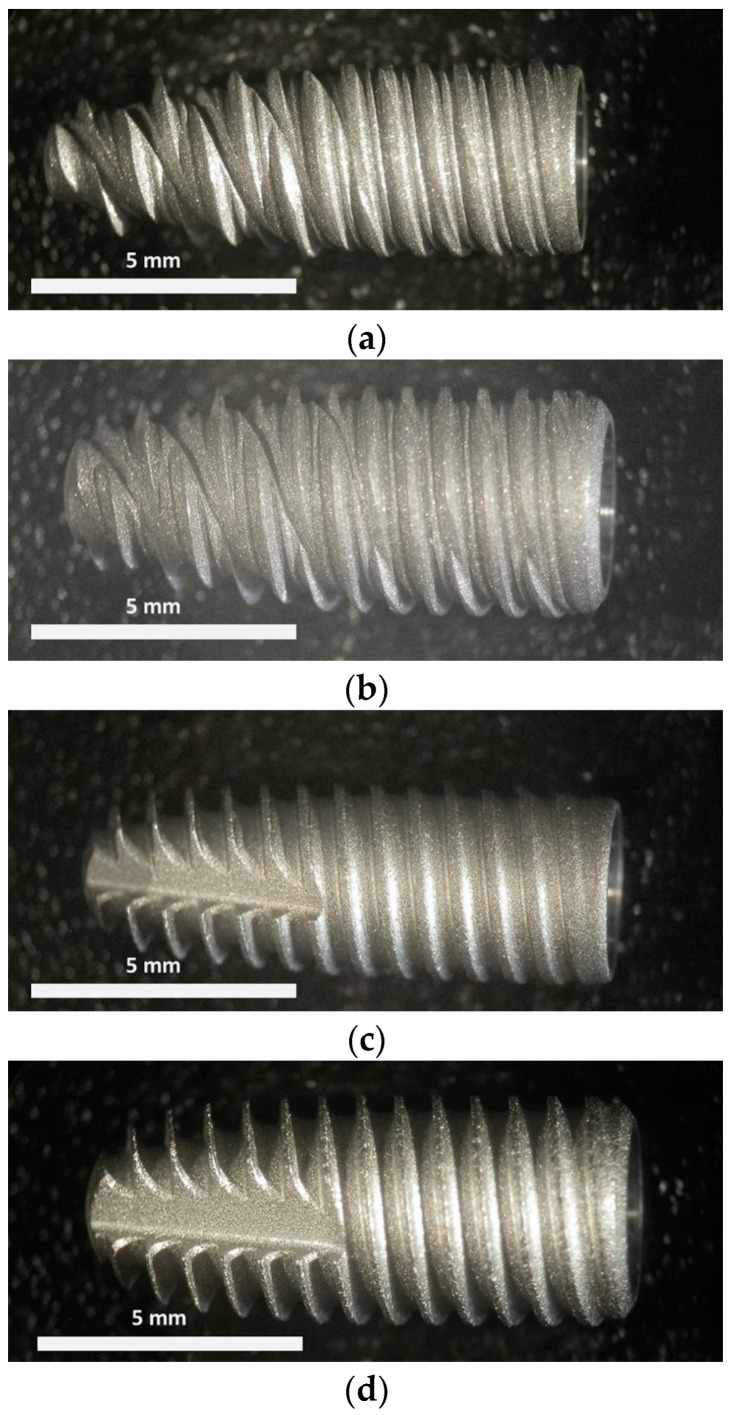
Macrographs of the dental implants used in this experimentation, specifically: (**a**) A, (**b**) B, (**c**) C, (**d**) D (see Table 1 for codes).

All the implants, as shown in Figure 1, had a conical shape; A and B were characterized by the presence of a double thread with a pitch of 0.7 mm each, while C and D by a single thread with a pitch of 0.7 mm. Finally, C and D had a kind of groove on the tip of the implant (see left side of the implants in Figure 1c,d). Fixtures A and B were made of titanium Grade 5, while fixtures C and D were made of CPTi.

The nominal compositions and the mechanical properties of the different titanium alloys are reported in Table 2; Table 3 [30].

The rugosimetric survey of the dental implants resulting from the different treatments, adopted as a function of the different base materials, was carried out through a Leica Definition Confocal Microscopy (DCM) 3 D (Leica Microsystems, Schweiz, AG-CH, Heerbrugg, Switzerland) equipped with LeicaScan and LeicaMap software (Leica Microsystems, Schweiz, AG-CH, Heerbrugg, Switzerland). To highlight the topographic features, a surface of 8.40 × 0.95 mm^2^ was acquired with a magnification of 10×, whereas to investigate the roughness impact, a reduced area of 1.27 × 0.50 mm^2^ was acquired with a magnification of 50×. In order to provide an accurate surface description, the roughness profiles were extrapolated from an acquired image and, successively, they were filtered using a cut-off of 80 µm according to ISO 4288 [31]. Surface texture can be described quantitatively by means of a certain number of parameters. These parameters represent different aspects of the surface, such as roughness, waviness, and shape. In order to predict the behavior of a component during its use, it is necessary to quantify the surface characteristics. This is possible by evaluating some parameters such as “amplitude”, “material”, “spacing”, and “hybrid” according to ISO 4287 [32].

The examined amplitude parameters were as follows: Ra as the arithmetical mean of the sums of roughness profile values; R_max_ as the maximum height between the highest peak and the deepest valley of the profile defined on the evaluation length; R_v_ as the maximum valley depth of the respective profile; R_p_, as the maximum peak height of the respective profile; R_z_, as the maximum height of the respective profile. In order to describe the profile symmetry, statistical parameters for height distribution were examined, i.e., R_sk_, a skewness measurement, which is an asymmetry index, and R_ku_, a Kurtosis measurement, which represents a “peakedness” index [33].

In addition, a surface morphology observation along with a study of the surface treatment impact on the chemical composition of the surface itself were carried out by means of a Hitachi TM3000 scanning electron microscope (SEM, Hitachi, Tokio, Japan) equipped with a National Instruments EDX microprobe (National Instruments Italy, Assago, Italy,). Prior to these observations, the implants were cleaned with acetone in an ultrasonic bath.

The statistical evaluation of the amplitude parameters for each implant was established by One-Way ANOVA (Fisher’s) analysis performed using the open source software Jamovi (Version 0.9, https://www.jamovi.org) with Tukey Post-Hoc Test for pair comparisons [34].

## 3. Results

**Figure 2:**
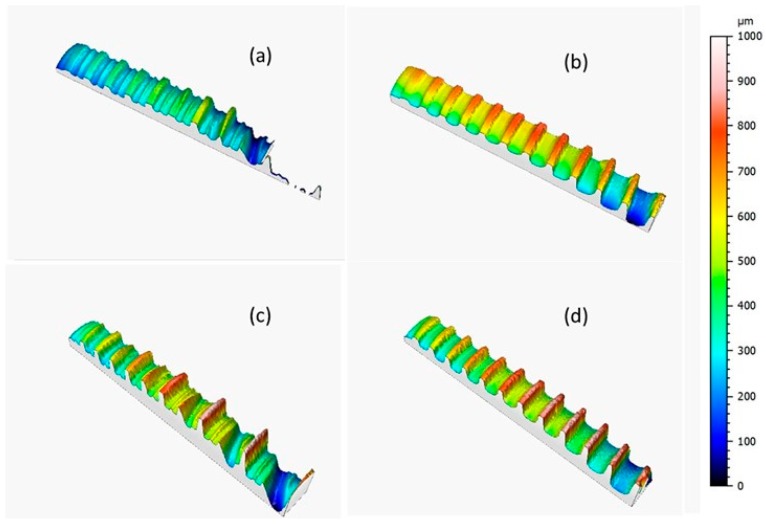
Topographies of the dental implants acquired with a Leica DCM3D microscope with 10× magnification: (**a**) A, (**b**) C, (**c**) B, and (**d**) D (scan size 8.4 × 0.95 mm^2^).

Figure 2 shows the implant topographies as acquired through Leica DCM3D and displayed at the same scale and scan size in order to analyze the implant morphologic features; the dental implants were screw-shaped with a single (Figure 2b,d) or double thread (Figure 2a,c).

**Figure 3:**
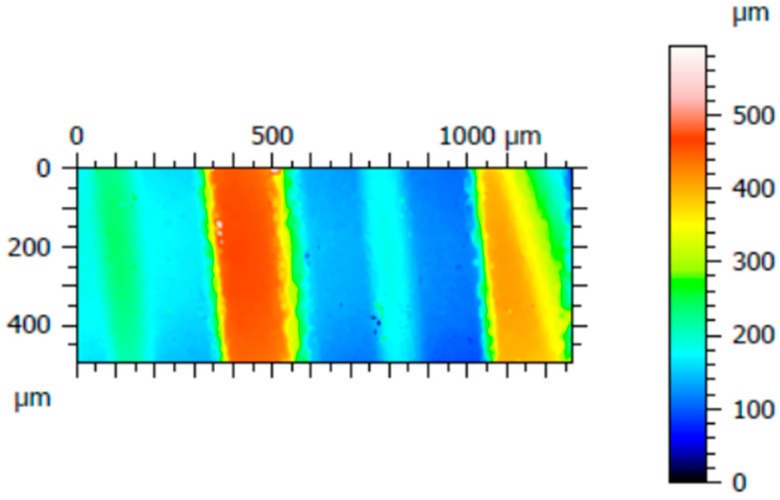
An acquired surface of implant D, with dimensions of 1.27 mm × 0.5 mm.

Three surfaces were acquired for each implant, from which three roughness profiles were measured. Figure 3 shows one of the three acquired areas of implant C; three roughness profiles were measured distanced 200 µm from each other, specifically, at 100 µm, 300 µm, and 500 µm starting from the left of the acquired surface. The same procedure was performed for the other implants.

**Figure 4:**
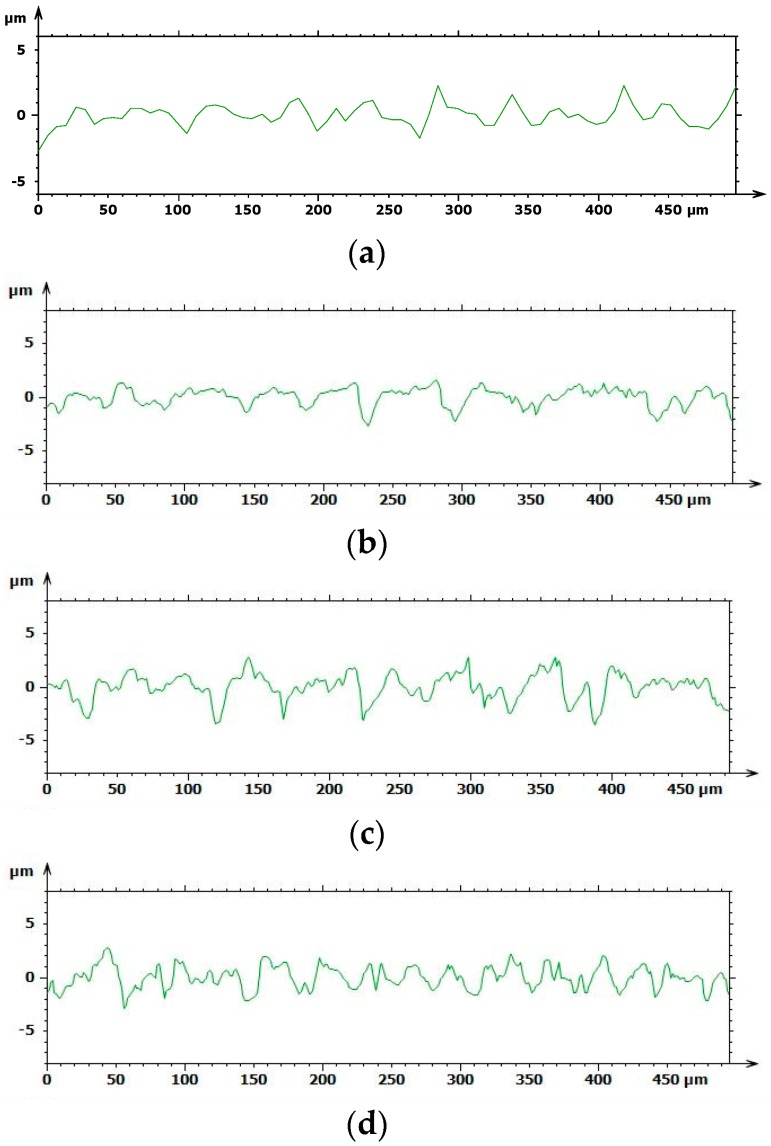
Roughness profiles of the implant surfaces extrapolated using Leica Map (**a**) A, (**b**) B, (**c**) C, and (**d**) D.

Based on the results above, the chemical treatment seemed to develop the coarsest surface with a spiky and sharp-cornered morphology, whilst a fluctuating morphology occurred with sandblasting, as shown in Figure 4. The A and B roughness profiles obtained with Leica Map appeared to be softer, more uniform, and flattened, whereas C and D showed more jagged and bristly profiles.

**Figure 5:**
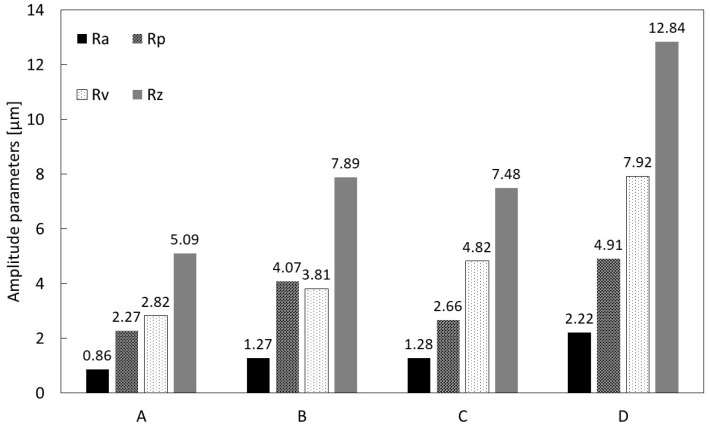
Roughness measurement of the dental implants estimated through the amplitude parameters R_a_, R_p_, R_v_, and R_z_ taken with Leica Map.

**Figure 6:**
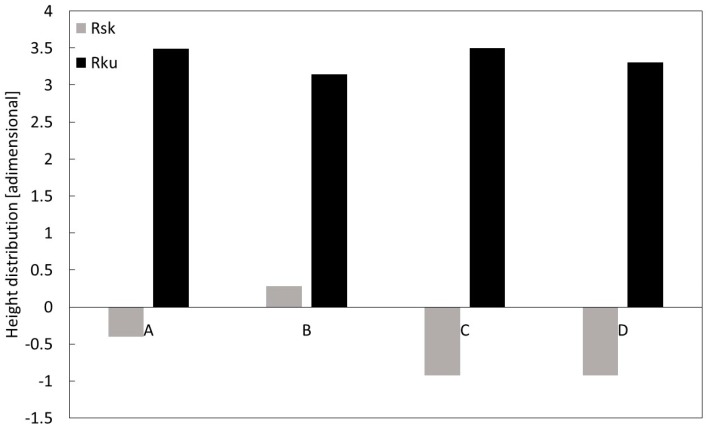
Height distribution of the dental implants’ roughness profiles estimated through the dimensional parameters R_sk_ and R_ku_, taken with Leica Map.

The average values of the amplitude parameters (R_a_, R_p_, R_v_, and R_z_) and height distribution parameters (R_ku_ and R_sk_) were calculated for each implant and are reported in Figure 5; Figure 6, respectively. Specifically, Figure 5 shows the highest R_a_ value was calculated for D, and the lowest for A, i.e., 0.86 µm versus 2.22 µm, respectively; a similar outcome was observed for the R_z_ values. In addition, in order to analyze the roughness profile shape, the statistical parameters of height distribution were examined, as shown in Figure 6.

The positive kurtosis value (R_ku_ > 0) and skewness values, which were approximately zero (R_sk_ ~ 0) in all A and B, suggested a fluctuating morphology with a high density of peaks, while C and D with negative kurtosis values indicated lower valley density.

**Figure 7:**
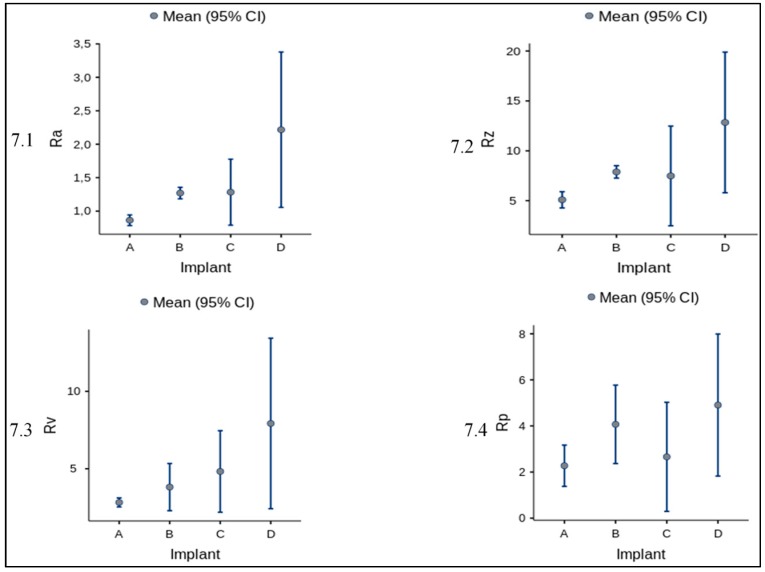
Mean and standard deviation of amplitude parameters for each implant. In figure are graphically represented the values reported in Table 4.

Furthermore, for amplitude parameters (R_a_, R_p_, R_v_, R_z_) the mean and standard deviation is reported in Figure 7 and Table 4 altogether with the ANOVA statistics (Table 5 and Table 6).

SEM observations were carried out on all examined dental implants using a Hitachi TM3000. For each implant, the images were acquired at two different magnification levels, 250× and 2500×; the former was applied to investigate the chemical composition, and the latter to observe surface details. The chemical compositions of the examined dental implants are reported in Table 7.

**Figure 8:**
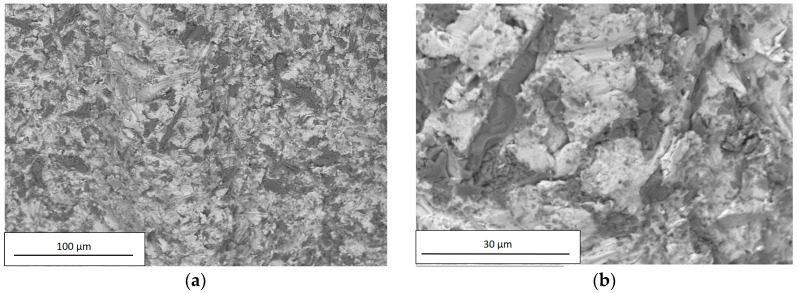
SEM observation of A, acquired using a Hitachi TM3000 with a magnification of (**a**) 250× and (**b**) 2500×.

**Figure 9:**
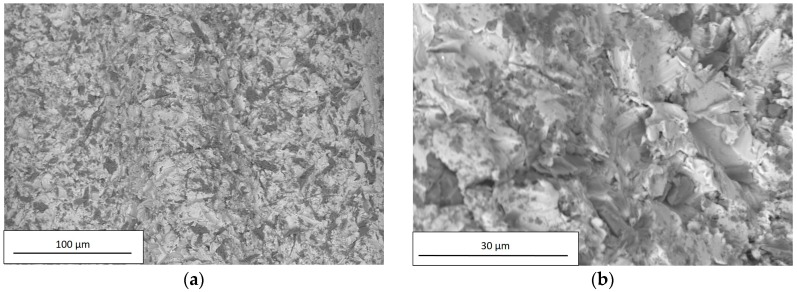
SEM observation of B, acquired using a Hitachi TM3000 with a magnification of (**a**) 250× and (**b**) 2500×.

As reported in Table 3, the high Al and O concentrations of A and B implant surfaces were likely due to residuals of Al_2_O_3_ used in the sandblasting process. The residuals are the dark particles visible in Figure 8 and Figure 9.

**Figure 10:**
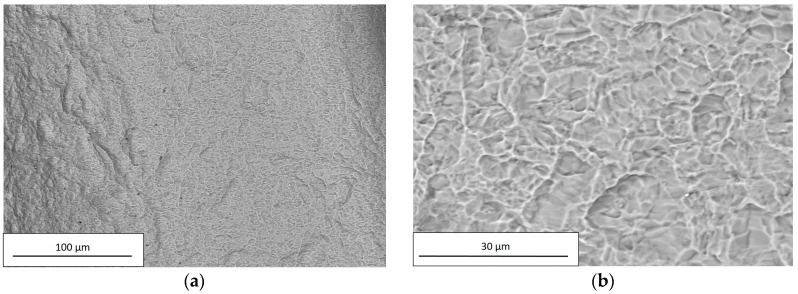
SEM observation of C, acquired using a Hitachi TM3000 with a magnification of (**a**) 250× and (**b**) 2500×.

**Figure 11:**
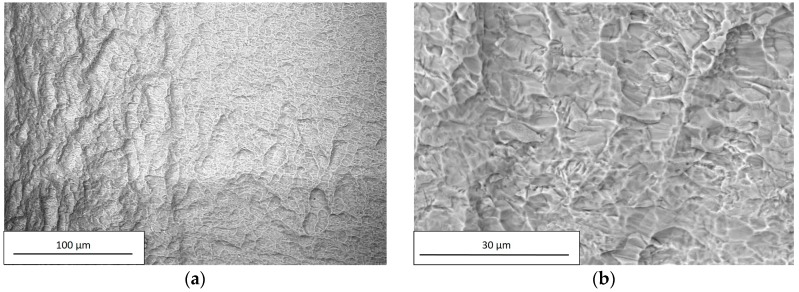
SEM observation of D, acquired using a Hitachi TM3000 with a magnification of (**a**) 250× and (**b**) 2500×.

Within the limits of the employed measuring technique, the composition of C and D implants did not reveal the presence of any residuals from the performed chemical treatment, as shown in Figure 10 and Figure 11 and in Table 7.

As the sandblasting process produced a surface with a symmetrical distribution of peaks and valleys thus acting in an undifferentiated way over the entire surface, the peaks and valleys were randomly distributed around the median plane, as confirmed by the values of R_sk_ in Figure 6. Furthermore, the presence of sharped edges as a result of plastic deformation (see Figure 8b and Figure 9b) accounted for the high values of R_ku_.

## 4. Discussion

Implant dentistry has become successful thanks to the discovery of the biological and mechanical properties of titanium [35]. Many studies reported that several factors, including implant design, surface topography (macro-, micro-, and nano-), wettability/energy, hydrophilicity or hydrophobicity, charge, and chemistry, appear to influence the inflammatory and regenerative phases which occur during osseointegration [36,37]. The implant macro-morphology has been shown to affect the osseointegration process. In general, the addition of implant threads might provide a potential positive contribution to bone implant contact (BIC). Some manufacturers have introduced double or triple threads parallel to each other, increasing the bone fixture contact compared to implants with only a single thread. From this point of view, the Kontact design is able to assure a larger bone interface than the Kontact S design. By increasing bone/fixture contact and modeling cell growth and osteoblast differentiation, the implant surface microtopography and microstructure play a key role in the peri-implant bone healing process [38]. Nowadays, there are no scientific indications that are able to define what a surface should be like, and dental implants are marketed without a clear definition of their surface characteristics. An optimal surface topography and roughness are still controversial, and different titanium surface treatments suggest that biomolecular adsorption, cell adhesion, as well as osteoblast maturation are promoted [39]. Microrough titanium surfaces have been produced using various procedures including sand blasting, acid-etching, or a combination of both with different clinical results [14]. Acid-etching is the most commonly used chemical method for surface treatment; some authors have indicated that thanks to this technique, an ideal superficial microtopography can be achieved to stimulate macrophages as well as the proliferation and a pro-angiogenic activity of endothelial cells immediately after implant placement [13,14]. Acid-etching is able to ensure an evident increase in BIC and initial osteoblast anchorage [14].

The mechanism of action of the chemical etching process is more complex and it is strongly influenced by different factors, mainly the initial topography of the surface. Particularly, the chemical treatment preserves the main features of the previous treatment. If the implants have been shaped by machining, their topography is characterized by the presence of deep and sharped grooves; after chemical treatment, those features are highlighted, the consequence of which is a surface with sharp and deep valleys. 

The rugosimetric survey of the dental implants resulting from the different treatments, was carried out through a Leica DCM 3 D equipped with LeicaScan and LeicaMap software. This confocal microscope with white light laser interferometry allowed for a high resolution study of the fixture surface finish. More specifically, confocal microscopy permitted the reconstruction of complex 3D surfaces, which could not be analyzed otherwise, through several optical sections and without any physical contact with the part. The full 3D mapping obtained also in recessed regions was not possible until few years ago, when stylus profilometers - or atomic force microscopy, on the higher resolution/smaller scan side-were only used [25].

In addition, the chemical treatment produces a localized corrosion, with the formation of pits spread on the entire metal surface, as can be seen from Figure 10b and Figure 11b.

Sandblasting seems to be able to increase CPTi roughness and the biomechanical features of a dental implant. This surface treatment also influences the primary stability of the fixture and promotes macrophagic, epithelial, and osteoblast cell surface adhesion [13]. The migration and function of macrophages are an important step towards the osseointegration process, these immune cells being able to remove necrotic debris from the surgical treatment and produce several growth factors thanks to their pro-osteogenic and pro-angiogenetic activities [14].

The titanium plasma spray (TPS) can be considered another well-established medical technology for improving surface roughness and wettability [40]. In general, a TPS surface structure is very rough (macro-roughness of up to 240 µm and micro-roughness approximately 40 μm) [41], as it is formed by overlapping droplets of solidified Ti [42], and is characterized by the occurrence of cavities, niches, clefts, and curved areas, resulting in a porous-like appearance. This special topography allows for an ingrowth of bone into the implant surface, as well as a direct structural and functional connection between living bone and the surface of a load-carrying implant (osseointegration) [43]. Such a connection reinforces the biomechanical interlocking of the bone with the implant [44]. 

SEM analysis of the examined implants revealed different chemical compositions and values of surface roughness in relation to different titanium grades, industrial superficial treatments, and fixture dimensions. The chemical composition and contamination at the interface is one aspect that significantly contributes to the biocompatibility of the implant in the vivo situation [9]. SEM observations suggested that the chemical treatment allowed for an CPT implant surface without contaminations. In contrast, in sandblasted implants, there could be a potential risk of surface contamination with the presence of blasting particle remnants [9]. All fixtures under review showed the presence of organic contamination such as carbon, which is unavoidable as atmospheric hydrocarbons are readily adsorbed by exposed titanium surfaces.

Acid-etching specimens presented a surface characterized by higher values of roughness parameters than the sandblasting specimens; the values of R_sk_, R_ku_, R_p_, and R_v_, confirm the above considerations.

The One-Way ANOVA analysis showed an overall statistically significant difference in group means (Table 5). To confirm where the differences occurred between groups, the Post hoc Tukey Test was performed (Table 6). As showed in Table 6, the main differences occurred between the measurements referred to implants A and D for all the parameters analysed (Ra, Rp, Rv, Rz). In particular, for Ra and Rz we can observe a statistically significant difference (with different degrees) for all paired implants, while for Rv only the pairs AD and BD show a statistically significant difference and for Rp exclusively the data for the pair AD is reported to be significant.

Many authors have revealed that the insertion of a rough fixture leads to increased titanium wear as opposed to smooth surfaces, especially during the screwing process of the implant placement procedure [25,45]. Van Staden has pointed out how different implant areas are in contact with the bone material at different times, in contrast to the expected uniform contact. When all the torque is concentrated on a smaller contact area versus the entire implant surface, a higher localized stress will result therefrom, which, in turn, can facilitate titanium release in a peri-implant bone [46]. Wear debris have been assumed to be one of the major factors responsible for aseptic implant loosening [47]. The real effects of metal and surface contaminant release in the osseointegration process are not yet fully understood, even though it is reported as a possible disturbing factor for bone remodeling balance and severe inflammatory and allergic reactions [48,49,50,51,52,53]. 

The implant surfaces examined showed different roughness levels in relation to the superficial treatment applied. The results highlighted that etched surfaces can be more easily damaged by the stress produced by the peri-implant bone during surgical implant placement. Therefore, long-term in vivo studies are necessary to evaluate any titanium toxic effect on tissue cells surrounding the implant surfaces.

## 5. Conclusions

On the basis of the experimental campaign carried out and discussed above, the following considerations can be drawn:

- Sandblasted dental implants showed R_a_ values lower than chemically etched implants, with a symmetrical distribution of peaks and valleys demonstrated by R_sk_ values next to zero.
- Chemically etched dental implants showed irregular roughness profiles characterized by the presence of deep valleys and high peaks.
- Sandblasted implants revealed the presence of residual Al_2_O_3_ on the surface as well as the existence of sharper edges as a result of plastic deformation.
- The topography of chemically etched implants was strongly influenced by the previous shaping processes. Furthermore, the chemical treatment produced a localized corrosion with the formation of pit spreads over the entire metal surface.
- The chemically etched implants bearing deeper valleys and high peak on the surface can be considered more vulnerable to titanium wear during implant placement than the sandblasted implants.

## Figures and Tables

**Table 1:** Main characteristics of the fixtures under examination.

Implant	Code	Material	Treatment	Diameter [mm]	Length [mm]	Thread
Kontact	A	Ti6Al4V	Sandblasted	3.6	10	Double
Kontact	B	Ti6Al4V	Sandblasted	4.2	10	Double
Kontact S	C	CP Ti	Chemically etched	3.6	10	Single
Kontact S	D	CP Ti	Chemically etched	4.2	10	Single

**Table 2:** Nominal weight composition of commercially pure titanium (CPTi) and Ti6Al4V used for the implant production.

Material	Weight %	Fe	V	Al	O	C	N	H	Other	Ti
**CP Ti**		0.20	0	0	0.18	0.10	0.03	0.015	0.30	99.175
**Ti6Al4V**	**Minimum**		3.50	5.50						87.725
	**Maximum**	0.40	4.50	6.75	0.20	0.80	0.03	0.015	0.30	91

**Table 3:** Mechanical properties of CPTi and Ti6Al4V.

Material	UTS [MPa]	YS0.2 [MPa]	ETM [GPa]	El [%]
Cp Ti	660	590	105	20
Ti6Al4V	950	880	113.8	14

**Table 4:** Group Descriptives.

	Implant	N	Mean	SD	SE
Ra	A	3	0.864	0.0319	0.0184
	B	3	1.270	0.0346	0.0200
	C	3	1.283	0.1986	0.1146
	D	3	2.217	0.4674	0.2698
Rp	A	3	2.273	0.3607	0.2083
	B	3	4.070	0.6851	0.3955
	C	3	2.660	0.9527	0.5500
	D	3	4.907	1.2413	0.7167
Rv	A	3	2.823	0.1159	0.0669
	B	3	3.813	0.6140	0.3545
	C	3	4.823	1.0599	0.6119
	D	3	7.923	2.2162	1.2795
Rz	A	3	5.093	0.3275	0.1891
	B	3	7.887	0.2499	0.1443
	C	3	7.483	2.0102	1.1606
	D	3	12.843	2.8367	1.6377

**Table 5:** One-Way ANOVA (Fisher’s).

	F	df1	df2	*p*
Ra	15.15	3	8	0.001 **
Rp	5.92	3	8	0.020 *
Rv	9.11	3	8	0.006 **
Rz	10.36	3	8	0.004 **

Note: * *p* < 0.05, ** *p* < 0.01.

**Table 6:** Tukey Post-Hoc Test.

		**Ra**					**Rp**				
		A	B	C	D		A	B	C	D	
A	Mean difference	—	−0.406	−0.4197	−1.353	***	—	−1.80	−0.387	−2.633	*
	*p*-value	—	0.281	0.259	<0.001		—	0.131	0.946	0.025	
B	Mean difference		—	−0.0133	−0.947	**		—	1.410	−0.837	
	*p*-value		—	1.000	0.008			—	0.272	0.658	
C	Mean difference			—	−0.933	**			—	−2.247	
	*p*-value			—	0.009				—	0.054	
D	Mean difference				—					—	
	*p*-value				—					—	
		**Rz**					**Rv**				
A	Mean difference	—	−0.990	−2.00	−5.10	**	—	−2.79	−2.390	−7.75	**
	*p*-value	—	0.777	0.288	0.005		—	0.280	0.396	0.003	
B	Mean difference		—	−1.01	−4.11	*		—	0.403	−4.96	*
	*p*-value		—	0.767	0.017			—	0.992	0.034	
C	Mean difference			—	−3.10				—	−5.36	*
	*p*-value			—	0.067				—	0.023	
D	Mean difference				—					—	
	*p*-value				—					—	

Note: * *p* < 0.05, ** *p* < 0.01, *** *p* < 0.001.

**Table 7:** Chemical compositions of the dental implants.

Element	A	B	C	D
	Weight [%]			
Titanium	39.7	47.3	100.0	100.0
Oxygen	42.5	37.8	/	/
Aluminum	15.9	12.6	/	/
Vanadium	1.9	2.3	/	/
